# Roles of *HOTAIR* in lung cancer susceptibility and prognosis

**DOI:** 10.1002/mgg3.1299

**Published:** 2020-05-11

**Authors:** Meng‐Meng Ren, Sen Xu, Yu‐Bo Wei, Juan‐Juan Yang, Ya‐Nan Yang, Shan‐Shan Sun, You‐Jie Li, Ping‐Yu Wang, Shu‐Yang Xie

**Affiliations:** ^1^ Department of Biochemistry and Molecular Biology Key Laboratory of Tumor Molecular Biology in Binzhou Medical University Binzhou Medical University YanTai P.R. China; ^2^ Department of Epidemiology Binzhou Medical University YanTai P.R. China; ^3^ Dongying People’s Hospital Binzhou Medical College Affiliated Teaching Hospital Dongying P.R. China

**Keywords:** *HOTAIR* gene, lung cancer, prognosis, SNPs, susceptibility

## Abstract

**Background:**

Long noncoding (lncRNA) single‐nucleotide polymorphisms (SNPs) are associated with the susceptibility to the development of various malignant tumors. The aim of this study was to investigate the roles of HOX transcript antisense intergenic RNA (*HOTAIR*) and its SNPs in lung cancer.

**Methods:**

Initially, the expression of *HOTAIR* in different tumors was investigated using the online Gene Expression Profiling Interactive Analysis (GEPIA) resource. Three SNPs (rs920778, rs1899663, and rs4759314) of *HOTAIR* were identified using the MassArray system. Following this, the relationship between these SNPs and susceptibility to lung cancer was investigated.

**Results:**

Expression of *HOTAIR* was found to increase in a variety of cancers, including nonsmall cell lung cancer (NSCLC). We found that the genotypes of these SNPs (rs920778, rs1899663, and rs4759314) were not significantly associated with lung cancer type, family history, lymph node metastasis, or lung cancer stage. In gender stratification, the results of rs920778 genotypes showed that, compared to genotype AA, the AG (OR = 0.344, 95% CI: 0.133–0.893, *p* = .028) and AG + GG (OR = 0.378, 95% CI: 0.153–0.932, *p* = .035) genotypes of rs920778 are protective factors against NSCLC in females. In smoking stratification, compared with AA of rs920778, the genotype AG + GG (OR = 0.507, 95% CI: 0.263–0.975, *p* = .042) was a protective factor against NSCLC in nonsmoking people. No statistical differences were observed in the classifications of rs1899663 and rs4759314 genotypes. Linkage disequilibrium analysis revealed a high linkage disequilibrium between the rs920778 and rs1899663 (D′ = 0.99, *r* ^2^ = .74), rs920778 and rs4759314 (D′ = 0.85, *r* ^2^ = .13), and rs1899663 and rs4759314 (D′ = 0.79, *r* ^2^ = .00).

**Conclusion:**

Our study demonstrated that *HOTAIR* expression increased in NSCLC, and that the genotypes of rs920778 could be useful in the diagnosis and prognosis of lung cancer.

## Graphical abstract

This study investigated the roles of *HOTAIR* and its SNPs in lung cancer. We found that *HOTAIR* increased in nonsmall cell lung cancer, and the genotypes of rs920778 are protective factors for female patients and nonsmoking people, which are useful for screening and prognosis for lung cancer.

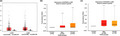

## 1 INTRODUCTION

Lung cancer, as a common malignant tumor, poses a serious threat to human health and is a grave public health problem worldwide, with more than one million people dying from lung cancer every year (Vachani, Sequist, & Spira, 2017). Although remarkable progress has been made in conventional treatments for lung cancer, many patients experience late‐stage diagnosis due to the absence of clear early‐stage symptoms. The prognosis of lung cancer is poor, the incidence of recurrence is high, drug resistance is common (Ma et al., 2017), and due to delayed diagnosis, the 5‐year survival rate is only 15% (Chen et al., 2016). With proper timely screening and the control of high‐risk factors in time, patients with lung cancer can be effectively treated or receive an optimistic prognosis; therefore, diagnosis and early treatment are key to prolonging the survival time of patients with lung cancer (Xue et al., 2015). There is consequently an urgent need for finding reliable biomarkers for screening and diagnosing lung cancer.

Lung cancer can be divided into small and nonsmall cell lung cancer (NSCLC). The latter accounts for approximately 85% of the total incidence of lung cancers, including lung adenocarcinoma (LUAD) and lung squamous cell carcinoma (LUSC). The main pathogenesis of NSCLC is epigenetic variations in chromatin, and the changes in chromatin modification products may alter the growth mode of cells and result in the loss of the original cellular characteristics (Scott, 2018).

Research from the last decade revealed that lncRNAs are involved in cancer development (Minotti, Agnoletto, Baldassari, Corra, & Volinia, 2018). LncRNA is a class of RNA with a length of more than 200 nucleotides that is rarely involved in encoding proteins. It has important molecular biological functions, such as transcriptional regulation, posttranscriptional regulation, translation regulation, and chromatin reconstruction, and plays a role in epigenetics (Wu et al., 2019). Increasing evidence shows that lncRNA is associated with the risk and prognosis of breast cancer, colorectal cancer, gastric cancer, and other malignant tumors (Xue et al., 2015). LncRNA expression is also correlated with tumor metastasis, late pathological stage, and prognoses in patients with lung cancer (Loewen, Jayawickramarajah, Zhuo, & Shan, 2014). Some researchers have suggested that lncRNAs could be significant biomarkers for cancer diagnosis and metastasis (Tong et al., 2015).

Single‐nucleotide polymorphisms (SNPs) and somatic mutation on lncRNAs might play a critical role in the pathogenesis of cancer, indicating a strong potential for further development of lncRNAs as biomarkers (Tong et al., 2015). HOX transcript antisense intergenic RNA (*HOTAIR*, HGNC ID *HGNC:33510*) with a length of 2,158 nt, is located in the homeobox C (HOXC) gene cluster on chromosome 12, and is found in the transcriptional D group of homeobox genes (Liu et al., 2013). *HOTAIR* was initially identified as lncRNA that interacts with polycomb inhibitory complex 2 (PRC2) and further inhibits the *HOXD* gene by binding to its 5′ domain. Its molecular scaffolds are regulated by inhibition of expression of PRC2 and lysine demethylase (Gupta et al., 2010). *HOTAIR* is highly expressed in various cancers, including lung cancer, and induces the proliferation and metastasis of cancer cells (Zhou et al., 2015). It can also induce tumorigenesis through epithelial mesenchymal conversion (Lee et al., 2016; Padua Alves et al., 2013; Tong et al., 2015). However, due to the limited understanding of its molecular mechanism, its role as a diagnostic marker in lung cancer is still unclear (Tan et al., 2018).

SNPs refer to the DNA sequence polymorphisms caused by single‐nucleotide variation at the genome level (Cooper, Smith, Cooke, Niemann, & Schmidtke, 1985). It is a part of genetic change and plays an important role in gene mutation. SNP variations existing in the coding or noncoding region of genes are associated with some diseases. For example, deleterious nonsynonymous SNPs in the tumor suppressor protein *TP53* gene affect the p53–estrogen receptor α interaction and are associated with breast cancer (Chitrala, Nagarkatti, Nagarkatti, & Yeguvapalli, 2019). Furthermore, the SNP rs915894 in *NOTCH4* gene may be a genetic marker for the prognosis of NSCLC in the Chinese population and may have an interactive relationship with epidemiologic factors (Xu, Lin, et al., 2019). In addition, an association was observed between SNPs in adiponectin gene + 276G/T and breast cancer incidence in postmenopausal women after adjustment for all other variables (Geriki et al., 2019). The AA genotype of SNP rs10889677 was significantly correlated with increased risk of colorectal cancer (Mosallaei et al., 2019). The toll‐like receptor gene 2 polymorphism, rs3804100, may be a potential prognostic biomarker for *Helicobacter pylori* infection‐independent gastric cancer (Zhao et al., 2019). Moreover, *HOTAIR* SNPs are associated with the susceptibility to various malignant tumors, including lung cancer (Bayram, Sumbul, Batmaci, & Genc, 2015; Guo et al., 2015; Pan et al., 2016; Xavier‐Magalhaes et al., 2017).

In order to explore the potential of *HOTAIR* as a diagnostic marker for lung cancer, we firstly investigated the expression of *HOTAIR* in different tumors using online Gene Expression Profiling Interactive Analysis (GEPIA) resources. Subsequently, the relationship between *HOTAIR* gene SNPs (rs920778, rs1899663, and rs4759314) and susceptibility to lung cancer was investigated. The correlation between SNP locus and gender and smoking was also tested to provide a basis for the early diagnosis of lung cancer, which will provide a scientific basis for lung cancer prognosis and therapy.

## 2 MATERIALS AND METHODS

### 2.1 Analyzing *HOTAIR* expression on GEPIA

The GEPIA server (http://gepia.cancer‐pku.cn/) allows the analysis of the prevalence of a gene signature in TCGA and GTEx samples (Tang et al., 2017). Here, the online resource was used to analyze the expression of *HOTAIR* gene in different tumors and the expression of genes in LUAD and LUSC. Kaplan–Meier survival analysis was utilized to examine the relationship between HOTAIR expression and the prognoses of patients with lung cancer.

### 2.2 Analyzing *HOTAIR* levels on UALCAN

UALCAN (http://ualcan.path.uab.edu/index.html) is an interactive website based on PERL‐CGI that analyzes publicly available cancer transcriptome data in the TCGA database. In this study uses the website UALCAN was used to analyze *HOTAIR* expression in LUAD and LUSC patients of different genders, or of different races. Kaplan–Meier survival analysis was utilized to estimate the relationship between *HOTAIR* expression and the prognoses of patients with LUAD and LUSC.

### 2.3 Sample collection

Blood samples from 196 patients were collected between January 1, 2015 and November 30, 2017, from the Dongying People's Hospital, Binzhou Medical College Affiliated Teaching Hospital. Patients in this study had been clinically diagnosed with lung cancer, but had not received radiotherapy or chemotherapy. Healthy control samples (*n* = 196) were collected from people who underwent physical examinations at the hospital during the same period, but who had no tumor and lung disease. All experiments were subject to approval by the Ethics Committee of Binzhou Medical University. Prior to inclusion, all the patients and controls provided written informed consent. The sample size was estimated by the formula of mismatching design by a formula described in the Supplemental Methods. Blood samples (2 ml each) were collected aseptically under aseptic condition and centrifuged at 2,000 *g* for 10 min (Eppendorf AG 22331). The centrifuged serum and blood cells were stored at −80°C.

### 2.4 DNA isolation and genotyping

Genomic DNA was isolated using a Mammalian Genomic DNA Extraction Kit according to the manufacturer's protocol (D0063, Beyotime Biotechnology). The extracted DNA was genotyped using a chip microarray detection system (Shanghai Ouyi Biomedical Technology Co., Ltd.). Briefly, three SNPs of *HOTAIR* gene were identified using the MassArray system (Agena iPLEXassay). Approximately 10–20 ng of genomic DNA were used for genotype analysis, and were amplified by a multiplex PCR reaction (primers are detailed in Table S1). The PCR products were then used for locus‐specific single‐base extension reactions, with resulting products were transferred to a 384‐element SpectroCHIP array. The alleles were discriminated by a mass spectrometry (Agena).

### 2.5 Statistical analysis

Basic data were analyzed using the Student′s *t* test. The chi‐squared test was used to compare gender, ancestry, residence, occupations, the gene frequency, smoking, alcohol consumption, family history, lymph node metastasis, and lung cancer staging. The genotype of the control group was expected to meet the Hardy–Weinberg equilibrium (HWE; *p* > .05). A logistic regression model was used to analyze the association between the gene polymorphisms and susceptibility to lung cancer, as well as the difference between the genotypes of different sites within each stratification. Linkage disequilibrium analysis and haplotype analysis were performed on three sites by using SHEsis software (http://analysis.bio‐x.cn/myAnalysis.php). Statistical analysis was conducted using software SPSS 22.0 software (IBM Corp.) with a two‐sided test, and the test level was *α* = 0.05.

## 3 RESULTS

### 3.1 *HOTAIR* expression in different types of cancers

**FIGURE 1:**
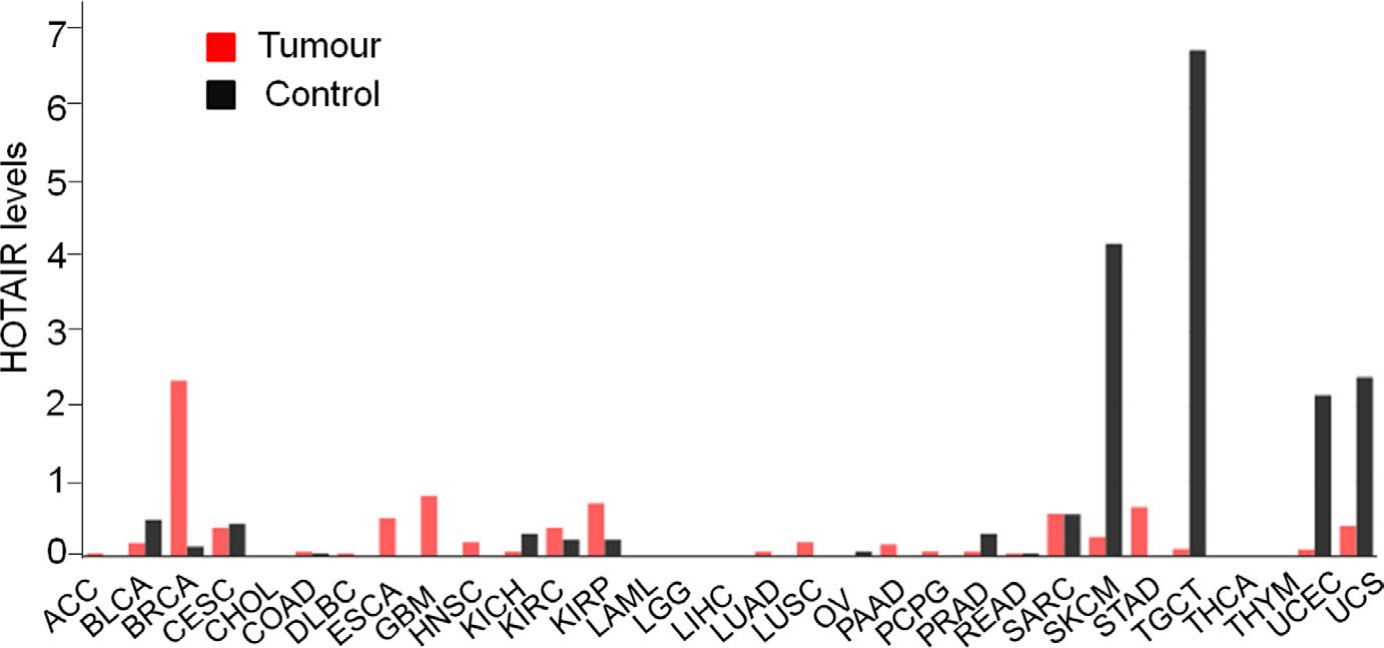
*HOTAIR* expression profile across all tumor samples and paired normal tissues. The height of bar represents the median expression of certain tumor type or control tissue. ACC, Adrenocortical carcinoma; BLCA, Bladder Urothelial Carcinoma; BRCA, Breast carcinoma; CESC, Cervical squamous cell carcinoma and endocervical adenocarcinoma; CHOL, Cholangiocarcinoma; COAD, Colon adenocarcinoma; DLBC, Lymphoid Neoplasm Diffuse Large B‐cell Lymphoma; ESCA, Esophageal carcinoma; GBM, Glioblastoma multiforme; HNSC, Head and Neck squamous cell carcinoma; KICH, Kidney Chromophobe; KIRC, Kidney renal clear cell carcinoma; KIRP, Kidney renal papillary cell carcinoma; LAML, Acute Myeloid Leukemia; LGG, Brain Lower Grade Glioma; LIHC, Liver hepatocellular carcinoma; LUAD, Lung adenocarcinoma; LUSC, Lung squamous cell carcinoma; OV, Ovarian serous cystadenocarcinoma; PAAD Pancreatic adenocarcinoma;, PCPG, Pheochromocytoma and Paraganglioma; PRAD, Prostate adenocarcinoma; READ, Rectum adenocarcinoma; SARC, Sarcoma; SKCM, Skin Cutaneous Melanoma; STAD, Stomach adenocarcinoma; TGCT, Testicular Germ Cell Tumors; THCA, Thyroid carcinoma; THYM, Thymoma; UCEC, Uterine Corpus Endometrial Carcinoma; UCS; Uterine Carcinosarcoma

*HOTAIR* gene is located in th*e HOXC* gene cluster on chromosome 12 [30], and plays an important role in the development of malignant cancers [16, 31, 32]. In order to further investigate the roles of *HOTAIR* in cancers, the GEPIA online resource (http://gepia.cancer‐pku.cn/) was used to study the expression levels of *HOTAIR* in different cancers. The results showed that *HOTAIR* is expressed in a variety of cancers, including breast cancer (BRCA), esophageal cancer (ESCA), glioblastoma multiforme (GBM), head and neck squamous cell carcinoma (HNSC), and kidney renal clear cell carcinoma (KIRC), etc. *HOTAIR* expression was significantly higher in the tissues of lung adenocarcinoma (LUAD), lung squamous cell carcinoma (LUSC), kidney renal papillary cell carcinoma (KIRP), stomach adenocarcinoma (STAD), BRCA, GBM, and HNSC, compared to that in the control tissues, which supports the oncogenic role of *HOTAIR* in these cancers (Figure 1).

### 3.2 Higher levels of *HOTAIR* in LUAD and LUSC

**FIGURE 2:**
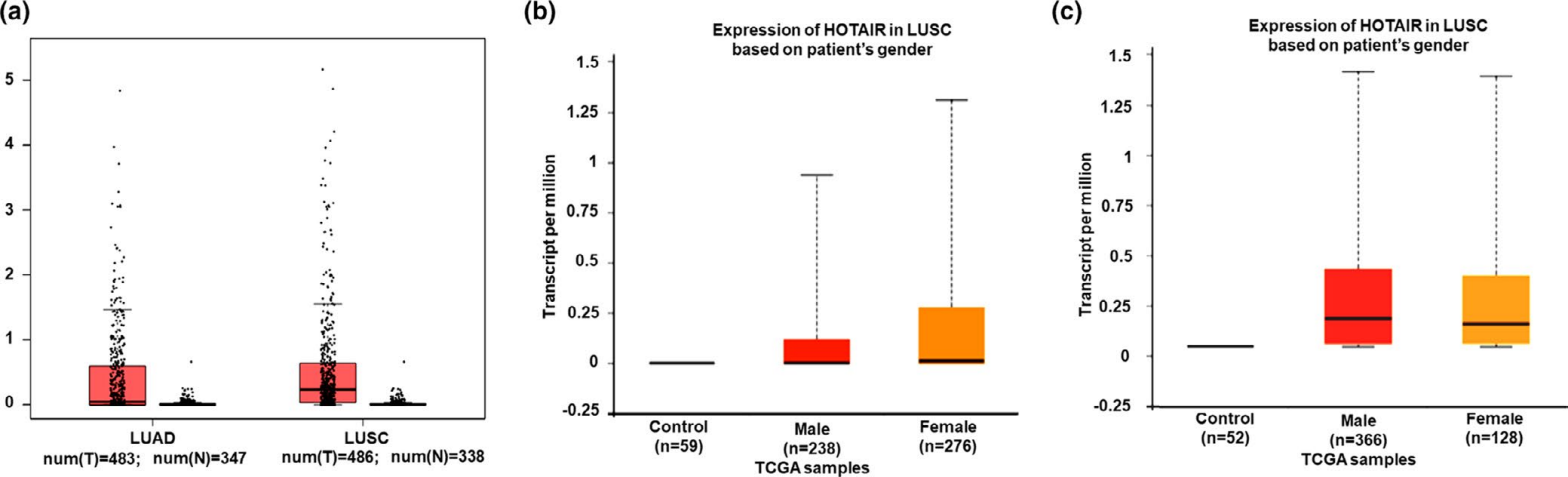
*HOTAIR* expression in patients with lung cancer. (a) *HOTAIR* expression in tissues of patients with LUAD or LUSC. (b) *HOTAIR* levels in male and female patients with LUAD. (c) *HOTAIR* in male and female patients with LUSC. *HOTAIR* expression increased in patients with LUAD or LUSC compared with the corresponding control samples. *HOTAIR* levels were higher both in male and female patients with LUAD or LUSC than those in control samples

*HOTAIR* is highly expressed in various cancers, and induces the proliferation and metastasis of these cancers. In order to investigate its roles in NSCLC, we next analyzed the expression levels of *HOTAIR* in LUAD and LUSC using the GEPIA sever. Results showed that *HOTAIR* expression was significantly increased in patients with LUAD (*n* = 483) compared to that in corresponding control samples (*n* = 347, *p* < .05). Significantly higher levels of *HOTAIR* were also found in patients with LUSC (*n* = 486) compared to controls (*n* = 338, *p* < .01, Figure 2a).

Using the UALCAN website (http://ualcan.path.uab.edu/analysis.html), we investigated whether gender could affect the expression of *HOTAIR* in LUAD or LUSC tissues. Results showed that the *HOTAIR* levels were higher in male (*n* = 238) and female patients (*n* = 276) with LUAD compare to control samples (*n* = 59, *p* < .001, Figure 2b). Levels of *HOTAIR* were also found to be much higher in male (*n* = 366) and female patients (*n* = 128) with LUSC compared to control samples (*n* = 52, *p* < .001, Figure 2c). However, there was on significant difference in *HOTAIR* expression between male and female patients with either LUAD (*p* = .058, Figure 2b) or LUSC (*p* = .319, Figure 2c).

### 3.3 *HOTAIR* expression and survival analysis of patients with lung cancer

**FIGURE 3:**
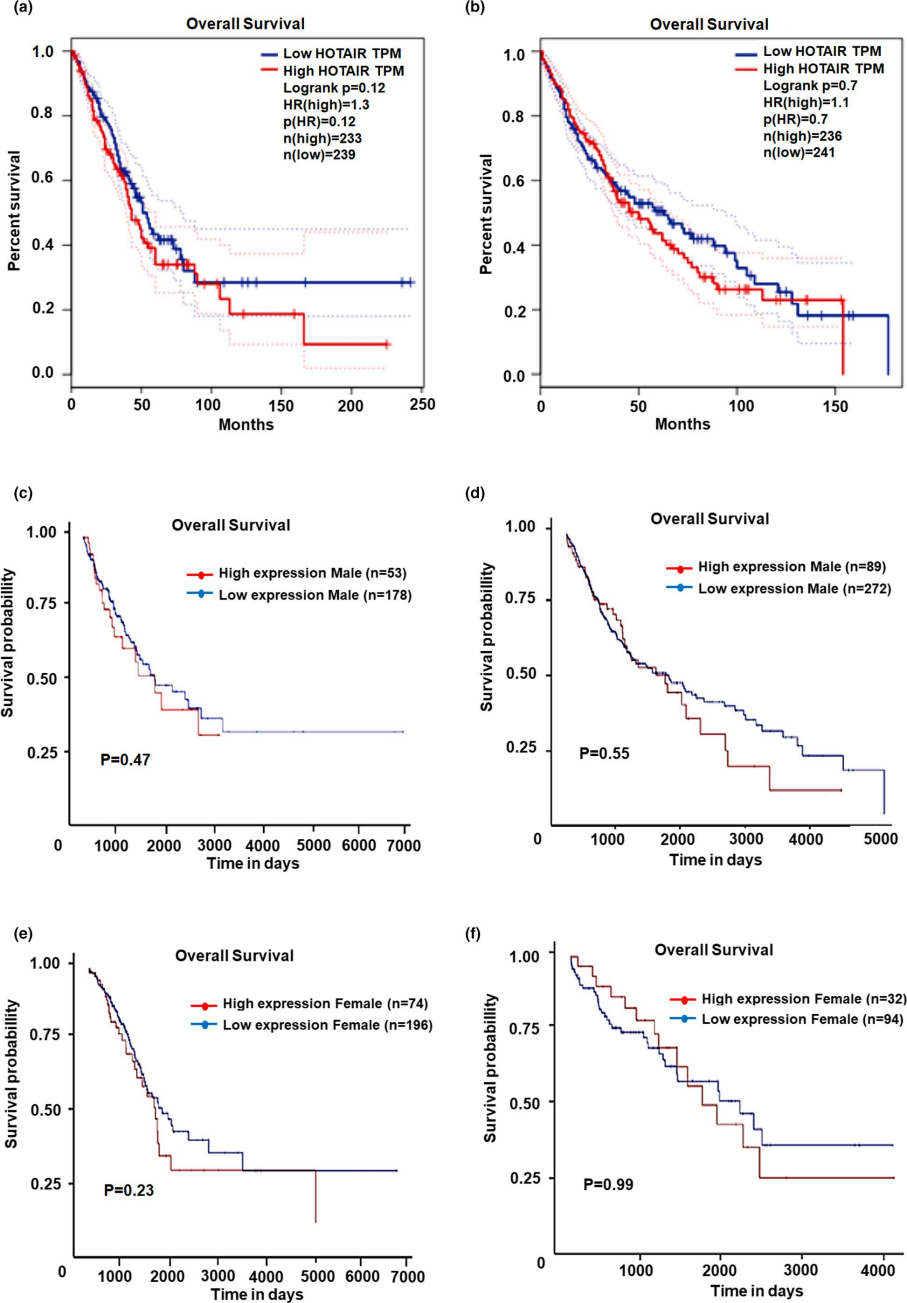
Relationship between total tumor expression and prognosis. (a) Patients with lung adenocarcinoma; (b) patients with lung squamous cell carcinoma; (c, d) Relationship between *HOTAIR* expression and prognosis of male patients with LUAD and LUSC, respectively; (e, f) Relationship between *HOTAIR* expression and prognosis of female patients with LUAD and LUSC, respectively. The overall survival of patients with LUAD or LUSC was not different between high and low expression of *HOTAIR*. Among patients with LUAD or LUSC, *HOTAIR* expression in male or female patients was not related with prognosis of patients

In order to investigate the effect of *HOTAIR* expression on the overall survival of patients with lung cancer, GEPIA software was used to analyze the relationship between the *HOTAIR* levels and prognoses in patients with LUAD or LUSC. The results showed that there was no significant difference between high and low expression of *HOTAIR* and the overall survival of patients with LUAD (*p* = .12, Figure 3a). Similarly, in patients with LUSC, no significant difference was found in survival analysis between patients with high and low expression of *HOTAIR* (*p* = .70, Figure 3b).

The relationship between *HOTAIR* expression and the prognoses of male or female patients with lung cancer was analyzed using UALCAN software. No significant differences were found between high and low expression of *HOTAIR* and disease prognoses in male patients with either LUAD (*p* = .47, Figure 3c) or LUSC (*p* = .55, Figure 3d), or disease prognoses in female patients with either LUSC (*p* = .23, Figure 3e) or LUSC (*p* = .99, Figure 3f).

### 3.4 *HOTAIR* SNPs and the susceptibility of patients with lung cancer

The abovementioned results indicate that the *HOTAIR* gene is highly expressed in various cancers, including lung cancer. Studies have shown that SNPs can affect the gene expression levels and is closely related to tumorigenesis (Wang et al., 2019). Therefore, we further explored the relationship between the *HOTAIR* gene SNPs (rs920778, rs1899663, and rs4759314) and the susceptibility to patients with lung cancer.

#### 3.4.1 Patient demographics

In order to investigate the relationship between the *HOTAIR* SNPs (rs920778, rs1899663, and rs4759314) and the susceptibility to lung cancer, we analyzed DNA from 196 cases of patients with NSCLC and 196 healthy controls. No statistical differences were observed in the age and gender between the case group and controls (*p* > .05). However, statistical differences were found between patients and the controls in terms of occupation, smoking, and alcohol consumption (*p* < .05, Table 1).

**TABLE 1:** Analysis of the basic situation of the case group and the control group

Variable	Control (%) (*n* = 196)	Case (%) (*n* = 196)	t/*χ* ^2^	*p*
Age (mean ± *SD*)	64.82 ± 10.00	64.32 ± 10.26	0.489	.741
Gender				
M	139 (70.9)	136 (69.4)	0.110	.682
F	57 (29.1)	60 (30.6)		
Occupation				
Employees of government	28 (14.3)	37 (18.9)	12.498	.002*
Farmer	86 (43.9)	110 (56.1)		
Others	82 (41.8)	49 (25.0)		
Smoking				
Not smoking < 180 sticks/year	137 (69.9)	97 (49.5)	23.820	＜.001*
≥180 sticks/year	53 (27.0)	98 (50.0)		
Quit smoking	6 (3.1)	1 (0.5)		
Alcohol consumption				
Not drinking	142 (72.4)	132 (67.3)	17.333	.001*
Small amount of alcohol (occasionally)	18 (9.2)	12 (6.1)		
At least once a week for more than half a year	28 (14.3)	52 (26.5)		
Stop drinking	8 (4.1)	0 (0.0)		

* *p* < .05.

#### 3.4.2 Three SNPs were detected and were in accordance with HWE equilibrium law

**FIGURE 4:**
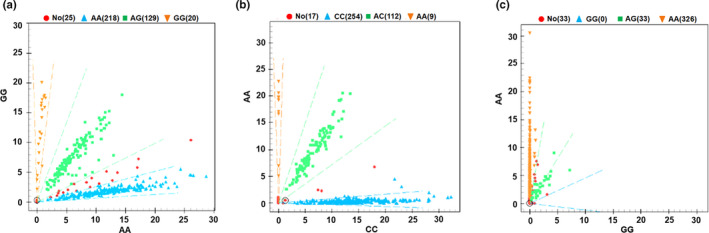
The genotypes of *HOTAIR* SNPs. (a) Dot plot of rs920778 genotype showed the number of AA, AG, and GG. (b) Dot plot of rs1899663 genotype showed the number of CC, AC, and AA. (c) Dot plot of rs4759314 genotype showed the number of GG, AG, and AA

Using a SNP MassArray system, we detected three SNP genotypes at different loci, including rs920778 in 183 controls and 184 cases, rs1899663 in 188 controls and 187 cases, and rs4759314 in 184 controls and 175 cases (Figure 4a–c).

Genetic variation of SNP rs920778, rs1899663, and rs4759314 within a population was analyzed using the population genetics HWE equilibrium law. These three SNP genotypes were all in accordance with the law of genetic inheritance (*p* > .05). The controls were well represented in these three genotypes (Table 2).

**TABLE 2:** Genotype Hardy–Weinberg equilibrium test analysis of the control group

SNP	Genotype	*n*	Composition ratio (%)	Estimated frequency	*χ* ^2^	*p*
rs920778	AA	104	56.8	104.8	0.109	.742
	AG	69	37.7	67.4		
	GG	10	5.5	10.8		
rs1899663	AA	5	2.7	6.5	0.533	.466
	AC	60	31.9	57.0		
	CC	123	65.4	124.5		
rs4759314	AA	168	91.3	168.3	0.380	.538
	AG	16	8.7	15.3		
	GG	0	0	0.3		

#### 3.4.3 *HOTAIR* SNPs and lung cancer risk analysis

Chi‐square tests were used to analyze alleles of *HOTAIR* SNP rs920778, rs1899663, and rs4759314 in patients with NSCLC and in healthy controls. Gene frequencies of rs920778 alleles A and G were 78.3% and 21.7% in the NSCLC group, and 75.7% and 24.3% in healthy controls, respectively. Gene frequencies of rs1899663 alleles A and C were 16.0% and 84.0% in NSCLC cases, and 18.6% and 81.4% in healthy controls, respectively. The gene frequencies of rs4759314 alleles A and G were 95.1% and 4.9% in NSCLC patients, and 95.7% and 4.3% in controls, respectively. The gene frequencies of the three loci were not significantly different between cases and controls (Table 3).

**TABLE 3:** Statistical analysis of gene frequency in the case group and control group

Genotype	Control *n* (%)	Case *n* (%)	*χ* ^2^	*p*	OR (95% CI)
rs920778					
A	277 (75.7)	288 (78.3)	0.688	.407	0.865 (0.613, 1.220)
G	89 (24.3)	80 (21.7)			
rs1899663					
A	70 (18.6)	60 (16.0)	0.867	.352	1.197 (0.819, 1.749)
C	306 (81.4)	314 (84.0)			
rs4759314					
A	352 (95.7)	333 (95.1)	0.106	.745	1.123 (0.558, 2.259)
G	16 (4.3)	17 (4.9)			

The genotypes of the rs920778, rs1899663, and rs4759314 in NSCLC cases and healthy controls were analyzed using chi‐square tests. Results demonstrated that the rs920778 locus genotypes AA, AG, GG, and AG + GG, the rs1899663 locus genotypes AA, AC, CC, and AC + CC, and the genotypes AA and AG of the rs4759314 locus between NSCLC and healthy controls were not significantly associated with the susceptibility to lung cancer (Table S2).

We further investigated whether the genotypes of locus rs920778, rs1899663, and rs4759314 are associated with lung cancer type, lymph node metastasis, and lung cancer stage, etc. Results show that AA, AG, and GG genotypes of rs920778 were not significantly associated with lung cancer type, family history, lymph node metastasis, or lung cancer stage (*p* > .05, Table S3). Similarly, the genotypes of rs1899663 (AA, AC, and CC) and of rs4759314 (AA and AG) were not significantly associated with lung cancer type, family history, lymph node metastasis, or lung cancer stage, respectively (*p* > .05, Tables 4 and 5).

**TABLE 4:** Stratified analysis of rs920778 and lung cancer risk

Variable	Control *n* (%)	Case *n* (%)	*p*	OR (95% CI)	Pa	aOR (95% CI)
*Gender*						
Female						
AA	30 (56.6)	40 (69.0)		1.000		1.000
AG	21 (39.6)	16 (27.6)	.173	0.571 (0.256, 1.277)	0.028	0.344 (0.133, 0.893)
GG	2 (3.8)	2 (3.4)	.780	0.725 (0.100, 5.633)	0.747	0.708 (0.087, 5.783)
AG + GG	23 (43.4)	18 (31)	.179	0.587 (0.270, 1.277)	0.035	0.378 (0.153, 0.932)
Male						
AA	74 (56.9)	74 (58.7)		1.000		1.000
AG	48 (36.9)	44 (34.9)	.743	0.917 (0.545, 1.543)	0.299	0.724 (0.394, 1.332)
GG	8 (6.2)	8 (6.3)	1.000	1.000 (0.356, 2.806)	0.518	0.653 (0.179, 2.377)
AG + GG	56 (43.1)	52 (41.2)	.770	0.929 (0.565, 1.525)	0.262	0.715 (0.398, 1.285)
*Smoking*						
Smoking						
AA	29 (52.7)	53 (57.6)		1.000		1.000
AG	24 (43.6)	33 (35.9)	.422	0.752 (0.376, 1.506)	0.300	0.651 (0.290, 1.464)
GG	2 (3.6)	6 (6.5)	.559	1.642 (0.311, 8.660)	0.884	1.150 (0.175, 7.553)
AG + GG	26 (47.2)	39 (42.4)	.564	0.821 (0.419, 1.607)	0.348	0.682 (0.307, 1.516)
Not smoking						
AA	75 (58.6)	61 (66.3)		1.000		1.000
AG	45 (35.2)	27 (29.3)	.308	0.738 (0.411, 1.324)	0.070	0.533 (0.269, 1.054)
GG	8 (6.3)	4 (4.3)	.444	0.615 (0.177, 2.139)	0.217	0.354 (0.068, 1.843)
AG + GG	53 (41.5)	31 (33.6)	.246	0.719 (0.412, 1.256)	0.042	0.507 (0.263, 0.975)
*Alcohol consumption*						
Drinking						
AA	31 (62.0)	38 (62.3)		1.000		1.000
AG	18 (36.0)	19 (31.1)	.714	0.861 (0.387, 1.917)	0.166	0.501 (0.189, 1.331)
GG	1 (2.0)	4 (6.6)	.301	3.263 (0.347, 30.715)	0.401	3.097 (0.222, 43.298)
AG + GG	19 (38.0)	23 (37.7)	.975	0.988 (0.457, 2.135)	0.280	0.597 (0.235, 1.520)
Not drinking						
AA	73 (54.9)	76 (61.8)		1.000		1.000
AG	51 (38.3)	41 (33.3)	.331	0.772 (0.458, 1.301)	0.128	0.630 (0.347, 1.143)
GG	9 (6.8)	6 (4.9)	.419	0.640 (0.217, 1.889)	0.161	0.399 (0.110, 1.442)
AG + GG	60 (44.1)	47 (38.2)	.264	0.752 (0.457, 1.239)	0.074	0.593 (0.335, 1.051)
*Occupation*						
Employees of government						
AA	14 (53.8)	21 (60.0)		1.000		1.000
AG	9 (34.6)	12 (34.3)	.833	0.889 (0.297, 2.663)	0.397	0.558 (0.145, 2.148)
GG	3 (11.5)	2 (5.7)	.406	0.444 (0.066, 3.010)	0.189	0.220 (0.023, 2.104)
AG + GG	12 (45.2)	14 (40.0)	.631	0.778 (0.279, 2.169)	0.222	0.458 (0.131, 1.603)
Farmer						
AA	43 (53.1)	65 (63.1)		1.000		1.000
AG	36 (44.4)	32 (31.1)	.089	0.588 (0.319, 1.085)	0.067	0.502 (0.240, 1.049)
GG	2 (2.5)	6 (5.8)	.414	1.985 (0.383, 10.293)	0.468	2.024 (0.301, 13.613)
AG + GG	38 (46.9)	38 (36.9)	.171	0.662 (0.366, 1.196)	0.127	0.576 (0.283, 1.171)
Others						
AA	47 (61.8)	28 (60.9)		1.000		1.000
AG	24 (31.6)	16 (34.8)	.779	1.119 (0.509, 2.458)	0.592	0.766 (0.288, 2.033)
GG	5 (6.6)	2 (4.3)	.647	0.671 (0.122, 3.695)	—	—
AG + GG	29 (38.2)	18 (39.1)	.915	1.042 (0.491, 2.209)	0.318	0.616 (0.238, 1.595)

Note

The logistic regression model was used to correct the age, ancestral home, past history, and the place of residence.

Pa and aOR were calculated by logistic regression with adjustment for age, gender, occupation smoking, and alcohol consumption.

**TABLE 5:** Stratified analysis of rs1899663 and lung cancer risk

Variable	Control *n* (%)	Case *n* (%)	*p*	OR (95% CI)	Pa	aOR (95% CI)
*Gender*						
Male						
AA	3 (2.2)	4 (3.1)		1.000		1.000
	42 (31.3)	37 (28.9)	0.603	0.661 (0.139, 3.147)	0.744	0.732 (0.113, 4.758)
CC	89 (66.4)	87 (68.0)	0.690	0.733 (0.159, 3.372)	0.917	0.907 (0.145, 5.686)
AC + CC	131 (97.7)	124 (96.9)	0.658	0.710 (0.156, 3.236)	0.858	0.847 (0.137, 5.238)
*Alcohol consumption*						
Drinking						
AA	1 (1.9)	1 (1.7)		1.000		1.000
AC	14 (26.4)	14 (23.3)	1.000	1.000 (0.057, 17.621)	0.843	0.697 (0.019, 25.081)
CC	38 (71.7)	45 (75.0)	0.906	1.184 (0.072, 19.576)	0.940	1.146 (0.034, 38.971)
AC + CC	52 (98.1)	59 (98.3)	0.929	1.135 (0.069, 18.598)	0.994	0.986 (0.031, 31.696)
Not drinking						
AA	4 (3.0)	3 (2.4)		1.000		1.000
AC	46 (34.1)	38 (29.9)	0.903	1.101 (0.232, 5.228)	0.519	1.828 (0.292, 11.464)
CC	85 (63.0)	86 (67.7)	0.701	1.349 (0.293, 6.209)	0.332	2.440 (0.403, 14.770)
AC + CC	127 (96.9)	123 (97.6)	0.764	1.262 (0.277, 5.753)	0.380	2.229 (0.372, 13.363)
*Occupation*						
Employees of government						
AA	2 (7.1)	2 (5.6)		1.000		1.000
AC	8 (28.6)	8 (22.2)	1.000	1.000 (0.112, 8.947)	0.622	1.921 (0.144, 25.713)
CC	18 (64.3)	26 (72.2)	0.725	1.444 (0.186, 11.221)	0.344	3.304 (0.278, 39.296)
AC + CC	26 (92.9)	34 (94.4)	0.795	1.308 (0.173, 9.911)	0.41	2.764 (0.245, 31.125)
Farmer						
AA	1 (1.2)	1 (1.0)		1.000		1.000
AC	30 (36.1)	31 (29.5)	0.982	1.033 (0.062, 17.282)	0.770	1.528 (0.089, 26.356)
CC	52 (62.7)	73 (69.5)	0.812	1.404 (0.086, 22.960)	0.573	2.252 (0.134, 37.821)
AC + CC	82 (98.8)	104 (99.0)	0.867	1.268 (0.078, 20.585)	0.636	1.970 (0.119, 32.661)

Note

The logistic regression model was used to correct the age, ancestral home, past history, and the place of residence.

Pa and aOR were calculated by logistic regression with adjustment for age, gender, occupation smoking, and alcohol consumption.

#### 3.4.4 Stratified analysis of *HOTAIR* SNPs and the risk of lung cancer

In order to further explore the relationship between SNPs and lung cancer risk, stratified analysis was carried out according to the gender, smoking, alcohol consumption, and occupation. Gender was divided into men and women, smoking is divided into smoking and nonsmoking, alcohol consumption divided into drinking and nondrinking, and occupations were divided into employees of government, farmers, and other occupations.

In gender stratification, the results of rs920778 genotypes showed that compared with genotype AA, the AG (OR = 0.344, 95% CI: 0.133–0.893, *p* = .028) and AG + GG (OR = 0.378, 95% CI: 0.153–0.932, *p* = .035) genotypes of rs920778 are protective factors against NSCLC in females (Table 4). In smoking stratification, compared with AA, the genotype AG + GG (OR = 0.507, 95% CI: 0.263–0.975, *p* = .042) was a protective factor against NSCLC in nonsmoking people (Table 4). In further classifications of rs920778, no statistical difference was observed (Table 4).

No statistical differences were observed in the classifications of rs1899663 and rs4759314 genotypes (Tables 5 and 6).

**TABLE 6:** Stratified analysis of rs4759314 and lung cancer risk

Variable	Control *n* (%)	Case *n* (%)	*p*	OR (95% CI)	Pa	aOR (95% CI)
*Gender*						
Female						
AA	49 (92.5)	50 (89.3)		1.000		1.000
AG	4 (7.5)	6 (10.7)	.569	1.470 (0.391, 5.531)	0.885	1.117 (0.250, 4.990)
Male						
AA	119 (90.8)	108 (90.8)		1.000		1.000
AG	12 (9.2)	11 (9.2)	.982	1.010 (0.428, 2.384)	0.159	0.448 (0.147, 1.370)
*Smoking*						
Smoking						
AA	48 (88.9)	82 (90.1)		1.000		1.000
AG	6 (11.1)	9 (9.9)	.816	0.878 (0.294, 2.619)	0.635	0.735 (0.206, 2.617)
Not smoking						
AA	120 (92.3)	76 (90.5)		1.000		1.000
AG	10 (7.7)	8 (9.5)	.638	1.263 (0.477, 3.342)	0.189	0.401 (0.102, 1.568)
*Alcohol consumption*						
Drinking						
AA	48 (94.1)	48 (87.3)		1.000		1.000
AG	3 (5.9)	7 (12.7)	.239	2.333 (0.569, 9.561)	0.828	1.217 (0.207, 7.140)
Not drinking						
AA	120 (90.2)	110 (91.7)		1.000		1.000
AG	13 (9.8)	10 (8.3)	.691	0.839 (0.354, 1.991)	0.197	0.504 (0.178, 1.429)
*Occupation*						
Employees of government						
AA	25 (89.3)	29 (93.5)		1.000		1.000
AG	3 (10.7)	2 (6.5)	.561	0.575 (0.089, 3.719)	0.342	0.304 (0.026, 3.539)
Farmer						
AA	74 (91.4)	92 (91.1)		1.000		1.000
AG	7 (8.6)	9 (8.9)	.949	1.034 (0.368, 2.909)	0.699	0.777 (0.215, 2.800)

Note

Pa and aOR were calculated by logistic regression with adjustment for age, gender, occupation smoking, and alcohol consumption.

#### 3.4.5 Linkage disequilibrium analysis of *HOTAIR* SNPs

**FIGURE 5:**
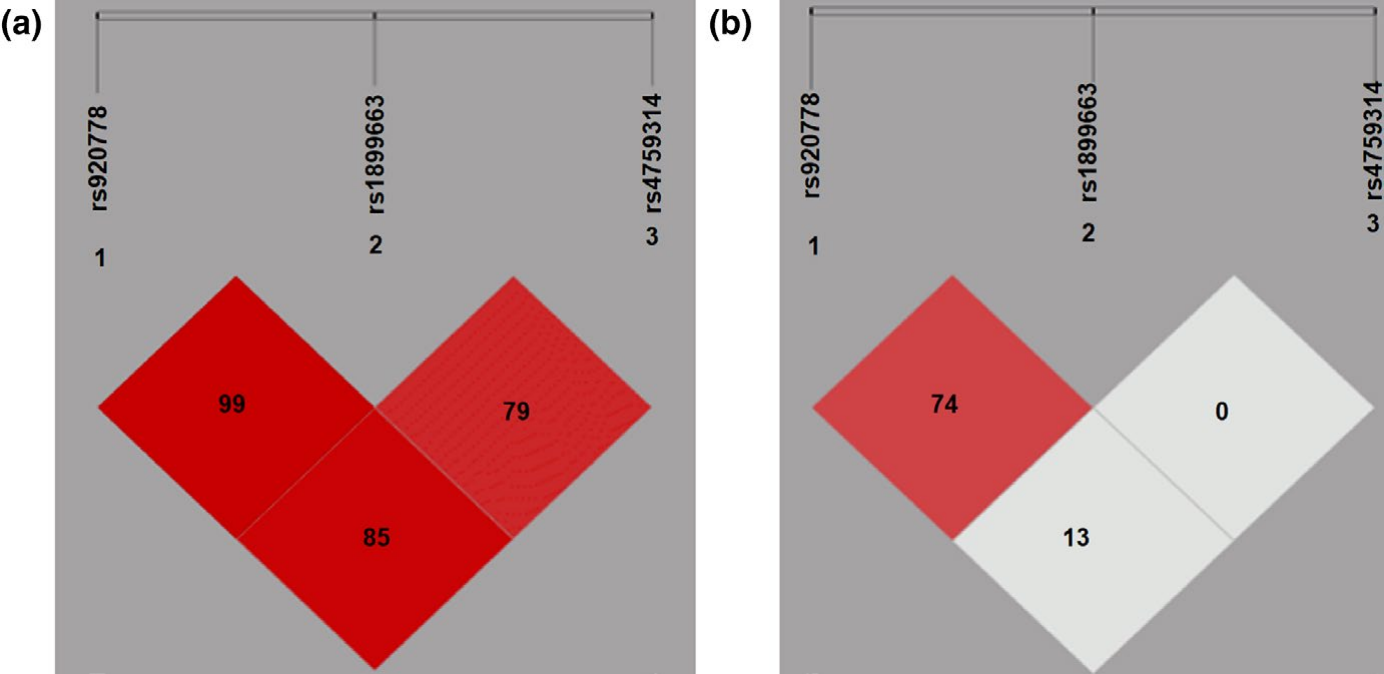
Results of linkage imbalance analysis. (a) Figure D′ value result diagram; (b) Figure *r* ^2^ value result diagram. The numbers represent a 100× increase in the values of D′ and *r* ^2^. A high linkage disequilibrium was found between rs920778 and rs1899663, or between rs920778 and rs4759314

In order to further explore the relationship between *HOTAIR* SNPs and the susceptibility to lung cancer, we used online software SHEsis to analyze the linkage disequilibrium of these three SNPs. The results of linkage disequilibrium analysis revealed a high linkage disequilibrium between the rs920778 and rs1899663 (D′ = 0.99, *r* ^2^ = .74), rs920778 and rs4759314 (D′ = 0.85, *r* ^2^ = .13), and rs1899663 and rs4759314 (D′ = 0.79, *r* ^2^ = .00) genotypes, indicating a high linkage disequilibrium between rs920778 and rs1899663. A linkage disequilibrium was also observed between rs920778 and rs4759314 (Figure 5).

The haplotypes of these three SNPs were studied using SHEsis software. The results showed that the common alleles in the lung cancer group and control group were A (rs920778), C (rs1899663), and A (rs4759314). The proportion of alleles in the lung cancer group and healthy control group was 78.3% and 76.4%, respectively; however, no significant difference was observed in the allele frequency between these two groups (Table S6).

#### 3.4.6 Bioinformatics analysis of SNP locus of *HOTAIR* gene

Following linkage disequilibrium analysis and haplotype analysis, these three loci were bioinformatically annotated using the public database HaploReg v4.1 (http://pubs.broadinstitute.org/mammals/haploreg/haploreg_v4.php). The three loci were all located on chromosome 12, and the affected bases of the three loci were all A. The reference base of site rs920778 and site rs4759314 was G, and the reference base of site rs1899663 was C. Some highly sensitive DNA regions were also found in these three loci. In addition, some change‐related motifs were observed at locus rs920778 and locus rs1899663, and they are also were quantitative trait loci for the expression of many genes (Table 7).

**TABLE 7:** HaploReg v4.1 database was used to annotate the functions of three sites of *HOTAIR* SNPs

Chr	pos (hg38)	variant	Ref	Alt	AFR freq	AMR freq	ASN freq	EUR freq	Promoter histone marks	Enhancer histone marks	DNAse	Motifs changed	Selected eQTL hits	GENCODE genes	dbSNP func annot
12	53966448	rs920778	G	A	0.31	0.58	0.76	0.69	12 tissues	11 tissues	13 tissues	DMRT4/5,THAP1	3 hits	*HOTAIR*	intronic
12	53967210	rs1899663	C	A	0.17	0.36	0.17	0.27	10 tissues	4 tissues	MUS	4 altered motifs	2 hits	*HOTAIR*	intronic
12	53968051	rs4759314	G	A	0.79	0.98	0.93	0.99	5 tissues	CRVX	7 tissues			*HOTAIR*	intronic

## 4 DISCUSSION

*HOTAIR* is one of the earliest studied lncRNAs, and is closely related to the lung cancer progression (Gupta et al., 2010; Herrera‐Solorio et al., 2017; Li et al., 2017). *HOTAIR* is a prognostic factor for various kinds of tumors (Kogo et al., 2011; Li et al., 2013; Zhuang et al., 2013), but the understanding of its role in tumor pathogenesis remains limited. In this case–control study, we explored the relationship between *HOTAIR* SNPs and susceptibility to lung cancer, and found that the AG and AG + GG genotypes of rs920778 are protective factors against NSCLC in females, and the genotype AG + GG was a protective factor against NSCLC in nonsmoking people. These data offer potential new tumor markers for screening and diagnosis of lung cancer by using public databases.

Data analysis has been become an important tool in determining cancer pathogenesis, in seeking treatment, and in identifying tumor markers (Minotti et al., 2018). The TCGA database is known as the most comprehensive database of cancer information worldwide, covering 39 types of cancer involving 29 cancer organs (X. Liu et al., 2019). The GTEx database contains more normal sample data than the TCGA database (Consortium, 2013). The GEPIA website mainly contains the data of the TCGA and GTEx databases (Ni et al., 2019), whereas the UALCAN website only contains the data of the TCGA database (Deng, Xu, & Wang, 2019). Therefore, this study first used different analytical functions of GEPIA and UALCAN to explore the expression of *HOTAIR* in the prognoses of patients with LUAD and LUSC, together with identifying effective biomarkers of LUAD and LUSC that will provide strong evidence for lung cancer prognosis.

We found that *HOTAIR* expression increased in LUAD and LUSC, and that the expression level was higher than in paracancerous tissues. We then analyzed the relationship between *HOTAIR* levels and prognoses of patients with LUAD and LUSC on the GEPIA website; no significant difference was observed in prognosis between patients with high expression levels and those with low expression patients, possibly due to the limited sample size and significant regional differences (Lv et al., 2019). The expression of *HOTAIR* in lung cancer patients of different genders was also analyzed. The expression level of *HOTAIR* in patients with LUAD or LUSC was higher than that in normal samples. However, no significant difference was observed between male and female patients.

SNP is the most typical type of genetic variant. The human genome probably contains approximately 10 million common SNPs (Mooney, 2005). SNPs that occur within the lncRNA transcripts can affect the structure and function of multiple RNA molecules (Cairns et al., 2019; Chen et al., 2019), whereas the presence of a SNP in the promoter region of a lncRNA could alter its expression level (Qin et al., 2019). In addition, somatic mutations that occur within lncRNAs exert important effects in cancer, and preliminary data are promising (Lu et al., 2019). Previous studies also revealed that SNPs in miR‐219‐1 (rs213210, rs421446, and rs107822) significantly affect the susceptibility and prognosis of NSCLC (Zheng et al., 2017).

Genetic variation of *HOTAIR* may affect its function and is related to the susceptibility of individuals to cancer development (Bayram, Ülger, et al., 2015; Xue et al., 2015). The minor alleles of *HOTAIR* rs4759314 and rs200349340 were significantly associated with pancreatic cancer susceptibility (Jiang et al., 2019). *HOTAIR* rs920778 was associated with esophageal cancer and esophageal squamous cell carcinoma risk (Tian, Liu, Liu, Zuo, & Chen, 2019). The T allele or TT genotype of *HOTAIR* polymorphisms could serve as a potential genetic marker for cancer risk, especially in Asians (Xu, Zhou, et al., 2019). *HOTAIR* SNPs rs12427129 and rs3816153 were associated with the risk of hepatocellular carcinoma (HCC) in dominant genetic models. Additionally, SNP‐environment interactions for rs12427129, rs3816153, and HBsAg status were found to enhance the risk of HCC (Zhang et al., 2019). Although, *HOTAIR* rs920778 and rs1899663 significantly increase susceptibility to lung cancer (Wang et al., 2018), the roles of *HOTAIR* SNPs in lung cancer still need to be further studied.

In this study, three SNPs of *HOTAIR* (rs920778, rs1899663, and rs4759314) were investigated in order to explore the relationship of these SNPs with the pathogenesis of lung cancer. Our results showed that no significant difference in the allele frequency of these three SNPs between lung cancer patients and healthy controls. The allele results of rs920778 is similar to Li's study (Li et al., 2018), but the allele results of rs4759314 is different from Li's study, which report that G allele carriers had a 2.598‐fold increased risk of developing lung cancer compared to A allele carriers (Li et al., 2018). This result may be due to regional differences and the size of the sample size (Chow et al., 2019). Interestingly, we found that AG or AG + GG genotypes of rs920778 were protective factors against lung cancer in female patients. In the smoking classification, AG + GG genotype of rs920778 was also a protective factor against lung cancer. In addition, significant differences were observed among the three genotypes of rs1899663 in the type of lung cancer. Similarly, Wang et al. found a significant association between *HOTAIR* SNP rs920778 and susceptibility to lung cancer (Wang et al., 2018).

In addition, rigorous literature screening and quality evaluation were used in this study. The relationship between SNPs in rs920778, rs1899663, and rs4759314 loci of the *HOTAIR* gene and susceptibility to lung cancer was analyzed from a genetics perspective.

In summary, this study investigated the roles of *HOTAIR* and its SNPs in lung cancer. We found that *HOTAIR* expression increased in NSCLC, and that the genotypes of rs920778 are protective factors in female patients and nonsmokers; this is useful for screening and prognosis for lung cancer.

## ETHICS STATEMENT

The experiments were approved by the Ethics Committee of Binzhou Medical University.

## PATIENT CONSENT FOR PUBLICATION

Prior to inclusion, all the patients and controls provided written informed consent.

## CONFLICT OF INTEREST

The authors declare that they have no conflict of interest concerning this article.

## AUTHORS' CONTRIBUTIONS

SYX and PYW conceived and designed the study. MMR, YBW, YNY, and SSS performed the experiments. JJY and YJL collected the samples. SX and SYX wrote the paper. SX, PYW, and SYX reviewed and edited the manuscript.

## Supporting information

Tables S1‐S6

Supplementary Material

## Data Availability

The data that support the findings of this study are available on request from the corresponding author. The genetic data are not publicly available due to privacy or ethical restrictions.
