# Supplementary material for: Roles of HOTAIR in lung cancer susceptibility and prognosis
Source: Mol Genet Genomic Med. 2020 May 11;8(7):e1299. doi: 10.1002/mgg3.1299 (PMC7336741; doi:10.1002/mgg3.1299)
Supplement: Supplementary file 1 — Tables S1‐S6 [file MGG3-8-e1299-s001.docx]

**Supplemental Tables 1-6**

**Supplemental Table 1. The primers used for the multiplex PCR reaction**

| SNP_ID | 2nd-PCRP | 1st-PCRP | UEP_SEQ |
| --- | --- | --- | --- |
| rs4759314 | ACGTTGGATGGTTCCTGCCTGGGCTTTTTC | ACGTTGGATGCCAGATGATCTGCTTGGAAG | TTATTAACTTGCATCCGC |
| rs1899663 | ACGTTGGATGCTGGTTCAATCTGATGCTCC | ACGTTGGATGGATCCCAGAGGCCATTTTTC | GCACAAGCCTCTAATTGTTGTCAC |
| rs920778 | ACGTTGGATGGGAGAGAAAATTAAACGTC | ACGTTGGATGGTGTACCGCCTTGTTTTCTG | TGACAGCTTAAATGTCTGAATGTTAC |

**Supplemental Table 2. Correlation analysis of genetic polymorphism and lung cancer susceptibility between the case group and control group**

| Variable | Control  n（%） | Case  n（%） | P | OR（95%CI） | Pa | aOR（95%CI） |
| --- | --- | --- | --- | --- | --- | --- |
| rs920778 |  |  |  |  |  |  |
| AA | 104（56.8） | 114（62.0） |  | 1.000 |  | 1.000 |
| AG | 69（37.7） | 60（32.6） | 0.298 | 0.793（0.513,1.227） | 0.238 | 0.764（0.489,1.195） |
| GG | 10（5.5） | 10（5.4） | 0.844 | 0.912（0.365,2.280） | 0.976 | 0.985（0.378,2.566） |
| AG+GG | 79（43.2） | 70（38.0） | 0.318 | 0.808（0.533,1.227） | 0.280 | 0.790（0.514,1.212） |
| rs1899663 |  |  |  |  |  |  |
| AA | 5（2.7） | 4（2.1） |  | 1.000 |  | 1.000 |
| AC | 60（31.9） | 52（27.5） | 0.909 | 1.083（0.276,4.247） | 0.929 | 1.067（0.254,4.479） |
| CC | 123（65.4） | 131（70.4） | 0.675 | 1.331（0.349,5.072） | 0.701 | 1.317（0.322,5.376） |
| AC+CC | 183（97.3） | 185（97.9） | 0.742 | 1.250（0.330,4.729） | 0.769 | 1.233（0.304,4.999） |
| rs4759314 |  |  |  |  |  |  |
| AA | 168（91.3） | 158（90.3） |  | 1.000 |  | 1.000 |
| AG | 16（8.7） | 17（9.7） | 0.739 | 1.130（0.552,2.313） | 0.727 | 1.139（0.548,2.368） |

*Note*. The logistic regression model was used to adjust the factory for age, gender, ancestral home, occupation, and place of residence. The genotypes of the rs920778, rs1899663, and rs4759314 in the case groups and the control group were analyzed by chi-square test. Pa and aOR were calculated by logistic regression with adjustment for age, gender, occupation smoking and alcohol consumption.

**Supplemental Table 3. Analysis of locus rs920778 genotype and lung cancer**

| Variable | AA  n（%） | AG  n（%） | GG  n（%） | χ2 | P |
| --- | --- | --- | --- | --- | --- |
| **Type of lung cancer** |  |  |  |  |  |
| Adenocarcinoma | 56（49.1） | 28（46.7） | 5（50.0） | 8.395 | 0.403 |
| Squamous cell carcinoma | 44（38.9） | 19（31.7） | 3（30.0） |  |  |
| Large cell carcinoma | 0（0.0） | 1（1.7） | 0（0.0） |  |  |
| Small cell carcinoma | 12（10.5） | 8（13.3） | 1（10.0） |  |  |
| Other | 2（1.8） | 4（6.7） | 1（10.0） |  |  |
| **Family history** |  |  |  |  |  |
| Have | 31（27.2） | 10（16.7） | 1（10.0） | 5.836 | 0.184 |
| No | 19（16.7） | 6（10.0） | 2（20.0） |  |  |
| Not clear | 64（56.1） | 44（73.3） | 7（70.0） |  |  |
| **Lymph node metastasis** |  |  |  |  |  |
| Have | 85（74.6） | 41（68.3） | 8（80.0） | 1.046 | 0.593 |
| No | 29（25.4） | 19（31.7） | 2（20.0） |  |  |
| **Lung cancer staging** |  |  |  |  |  |
| I-II | 38（33.3） | 24（40.0） | 2（20.0） | 1.789 | 0.409 |
| III-IV | 76（66.7） | 36（60.0） | 8（80.0） |  |  |

**Supplemental Table 4. Analysis of rs1899663 genotype frequency and lung cancer status**

| Variable | AA  n（%） | AC  n（%） | CC  n（%） | χ2 | P |
| --- | --- | --- | --- | --- | --- |
| **Type of lung cancer** |  |  |  |  |  |
| Adenocarcinoma | 1（25.0） | 27（51.9） | 61（46.6） | 19.017 | 0.008 |
| Squamous cell carcinoma | 3（75.0） | 10（19.2） | 55（42.0） |  |  |
| Large cell carcinoma | 0（0.0） | 1（1.9） | 0（0.0） |  |  |
| Small cell carcinoma | 0（0.0） | 9（17.3） | 12（9.2） |  |  |
| Other | 0（0.0） | 5（9.6） | 3（2.3） |  |  |
| **Family history** |  |  |  |  |  |
| Have | 1（25.0） | 6（11.5） | 34（26.0） | 5.691 | 0.171 |
| No | 0（0.0） | 7（13.5） | 20（15.3） |  |  |
| Not clear | 3（75.0） | 39（75.0） | 77（58.8） |  |  |
| **Lymph node metastasis** |  |  |  |  |  |
| Have | 3（75.0） | 37（71.2） | 98（74.8） | 0.450 | 0.879 |
| No | 1（25.0） | 15（28.8） | 33（25.2） |  |  |
| **Lung cancer staging** |  |  |  |  |  |
| I-II | 1（25.0） | 18（34.6） | 45（34.4） | 0.156 | 0.925 |
| III-IV | 3（75.0） | 34（65.4） | 86（65.6） |  |  |

**Supplemental Table 5. Analysis of rs4759314 genotype frequency and lung cancer status**

| Variable | AA  n（%） | AG  n（%） | χ2 | P |
| --- | --- | --- | --- | --- |
| **Type of lung cancer** |  |  |  |  |
| Adenocarcinoma | 79（50.0） | 7（41.2） | 2.482 | 0.650 |
| Squamous cell carcinoma | 53（33.5） | 8（47.1） |  |  |
| Large cell carcinoma | 1（0.6） | 0（0.0） |  |  |
| Small cell carcinoma | 18（11.4） | 1（5.9） |  |  |
| Other | 7（4.4） | 1（5.9） |  |  |
| **Family history** |  |  |  |  |
| Have | 36（22.8） | 5（29.4） | 0.499 | 0.870 |
| No | 22（13.9） | 2（11.8） |  |  |
| Not clear | 100（63.3） | 10（58.8） |  |  |
| **Lymph node metastasis** |  |  |  |  |
| Have | 121（76.6） | 10（58.8） | 2.572 | 0.109 |
| No | 37（23.4） | 7（41.2） |  |  |
| **Lung cancer staging** |  |  |  |  |
| I-II | 52（32.9） | 8（47.1） | 1.363 | 0.243 |
| III-IV | 106（67.1） | 9（52.9） |  |  |

**Supplemental Table 6. Results of haplotype analysis at three sites**

| rs920778 | rs1899663 | rs4759314 | Case  n（%） | Control  n（%） | χ2 | Fisher’s P | Pearson’s P | OR（95%CI） |
| --- | --- | --- | --- | --- | --- | --- | --- | --- |
| A | C | A | 263.2（78.3） | 269（76.4） | 0.816 | 0.366 | 0.366 | 1.181（0.823,1.697） |
| G | A | A | 56.8（16.6） | 67（19.0） | 0.583 | 0.445 | 0.445 | 0.858（0.580,1.270） |
| G | C | G | 13.0（3.9） | 16（4.5） | 0.167 | 0.682 | 0.683 | 0.856（0.405,1.808） |
| A | C | G | 3.8（1.1） |  |  |  |  |  |
| G | A | G | 0.2（0.1） |  |  |  |  |  |

Note: All alleles with a gene frequency less than 0.03 will be ignored in the analysis.
