# Supplementary material for: Roles of HOTAIR in lung cancer susceptibility and prognosis
Source: Mol Genet Genomic Med. 2020 May 11;8(7):e1299. doi: 10.1002/mgg3.1299 (PMC7336741; doi:10.1002/mgg3.1299)
Supplement: Supplementary file 2 — Supplementary Material [file MGG3-8-e1299-s002.docx]

**Supplemental Materials and Methods**

**Sample size design**

The sample size was estimated by the formula of mismatching design, and the number of cases was equal to that of the control group. The formula was as follows, and the sample size was calculated.

$n=\frac{2\overline{p}\overline{q}\left(z_{\alpha}+z_{\beta}\right)}{\left(p_{1}-p_{0}\right)^{2}}^{2}$ $p_{1}=\frac{p_{0}RR}{1+P_{0}\left(RR-1\right)}$,

$\overline{\text{p}}=0.5\times\left(p_{1}+p_{0}\right)$. $\overline{\text{q}}=1-\overline{p}$
